# Novel Approaches in the Immunotherapy of Multiple Sclerosis: Cyclization of Myelin Epitope Peptides and Conjugation with Mannan

**DOI:** 10.3390/brainsci11121583

**Published:** 2021-11-29

**Authors:** John M. Matsoukas, Irene Ligielli, Christos T. Chasapis, Konstantinos Kelaidonis, Vasso Apostolopoulos, Thomas Mavromoustakos

**Affiliations:** 1 NewDrug PC, Patras Science Park, 265 04 Platani, Greece; k.kelaidonis@gmail.com; 2 Institute for Health and Sport, Victoria University, Melbourne, VIC 3030, Australia; Vasso.Apostolopoulos@vu.edu.au; 3 Department of Physiology and Pharmacology, Cumming School of Medicine, University of Calgary, Calgary, AB T2N 4N1, Canada; 4 Department of Chemistry, University of Athens, 157 72 Athens, Greece; eir.ligielli@gmail.com; 5 NMR Facility, Instrumental Analysis Laboratory, Institute of Chemical, School of Natural Sciences, University of Patras, 265 04 Patras, Greece; cchasapis@upatras.gr; 6 Engineering Sciences, Foundation for Research and Technology, Hellas (FORTH/ICE-HT), 265 04 Patra, Greece; 7 Australian Institute for Musculoskeletal Science (AIMSS), Immunology Program, Melbourne, VIC 3021, Australia

**Keywords:** myelin epitope peptides, MBP87-99, MOG35-55, PLP139-151, Cyclization, conjugation with mannan, vaccines

## Abstract

Multiple Sclerosis (MS) is a serious autoimmune disease. The patient in an advanced state of the disease has restrained mobility and remains handicapped. It is therefore understandable that there is a great need for novel drugs and vaccines for the treatment of MS. Herein we summarise two major approaches applied for the treatment of the disease using peptide molecules alone or conjugated with mannan. The first approach focuses on selective myelin epitope peptide or peptide mimetic therapy alone or conjugated with mannan, and the second on immune-therapy by preventing or controlling disease through the release of appropriate cytokines. In both approaches the use of cyclic peptides offers the advantage of increased stability from proteolytic enzymes. In these approaches, the synthesis of myelin epitope peptides conjugated to mannan is of particular interest as this was found to protect mice against experimental autoimmune encephalomyelitis, an animal model of MS, in prophylactic and therapeutic protocols. Protection was peptide-specific and associated with reduced antigen-specific T cell proliferation. The aim of the studies of these peptide epitope analogs is to understand their molecular basis of interactions with human autoimmune T-cell receptor and a MS-associated human leucocyte antigen (HLA)-DR2b. This knowledge will lead the rational design to new beneficial non-peptide mimetic analogs for the treatment of MS. Some issues of the use of nanotechnology will also be addressed as a future trend to tackle the disease. We highlight novel immunomodulation and vaccine-based research against MS based on myelin epitope peptides and strategies developed in our laboratories.

## 1. Introduction

Multiple Sclerosis (MS) is a serious systemic demyelinating disease that leads to the partial paralysis of the patient who needs the assistance of medicare in order to survive with low quality of life. The need for an effective treatment in the form of medication or vaccine is more urgent than ever before [1,2,3,4,5]. This chronic inflammatory and neurodegenerative disease is initiated by autoreactive T helper (Th) cells and affects approximately 2.5 million people worldwide. Thus, there is an urgent need to develop effective treatments. Advances in the immunotherapy of MS have been recently reported in excellent reviews [6,7,8,9]. The pathogenesis of MS has been extensively studied over the last years, which shows a complex immunological involvement. Myelin epitopes have been identified as a target for autoimmune CD4+ T cells and antibodies, and much focus has been around the modulation of Th1 pro-inflammatory autoreactive CD4+ T cells against myelin epitopes, namely myelin basic protein (MBP), proteolipid protein (PLP), and myelin oligodendrocyte glycoprotein (MOG) [10,11,12,13,14,15,16,17,18].

**Figure 1:**
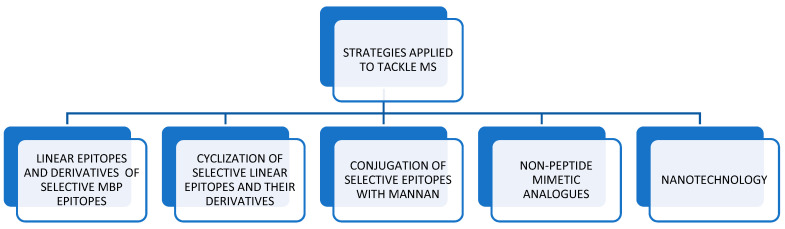
Immune modulation strategies used to tackle MS.

Herein, we summarize the efforts utilized to develop novel approaches to manage MS, which are primarily efforts of immune modulation via the synthesis of selective peptide epitopes of MBP, PLP, and MOG with or without conjugation to mannan. In addition, we provide the efforts applied for rational design to synthesize non-peptide mimetic analogues, as well as some nanotechnology approaches. We review evidence on how the knowledge of the molecular basis of interactions of the studied peptide analogues in complex with the major histocompatibility (MHC) class II molecule (HLA-DR2b) and its interaction with the T-cell receptor (TCR) can lead to the rational design of new therapeutic non-peptide mimetic analogs for the treatment of MS. The utilized strategies to approach the disease are (Figure 1):

(a) Use linear MBP, PLP, and MOG epitopes with or without amino acid mutations to gain insights into the molecular basis of the disease. This information is aided by nuclear magnetic resonance (NMR) studies and structural information of the epitope ligand bound to the MHC [19];
(b) Perform cyclization of these epitopes to increase their stability [20,21,22,23,24,25,26];
(c) Conjugate selective epitopes with mannan [27,28,29,30];
(d) Synthesis of non-peptide mimetic analogs to decrease their flexibility and increase their therapeutic index;
(e) Apply nanotechnology as a means of antigen delivery [31,32,33,34,35,36,37,38,39]

## 2. Applied Strategies Utilized against MS

### 2.1. Linear Epitopes and Derivatives of Selective MBP Epitopes

**Figure 2:**
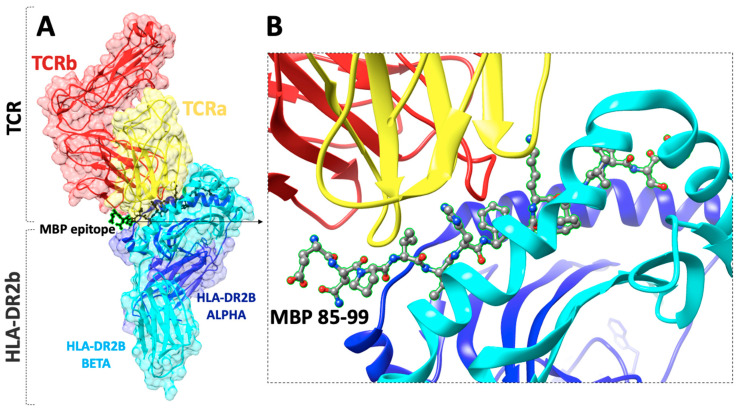
(**A**) The X-ray structure (pdb: 1YMM) of a human autoimmune TCR bound to a myelin basic protein (MBP_85–99_) peptide and a MS-associated MHC class II molecule (HLA-DR2b), (**B**) close view of the docking site of (MBP_85–99_) peptide.

**Figure 3:**
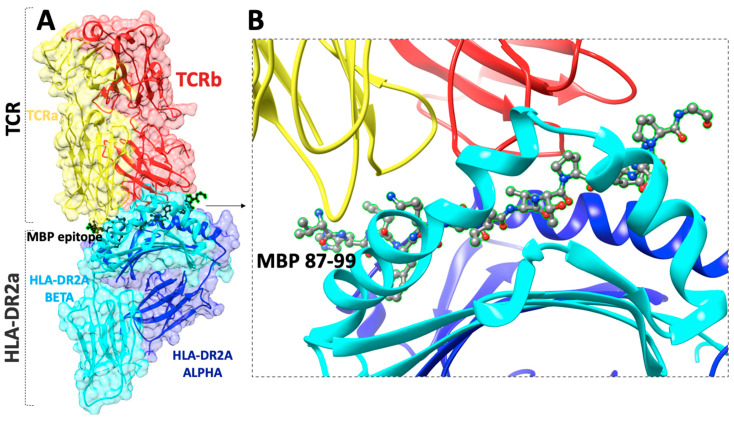
(**A**) The X-ray structure (pdb: 1ZGL) of a human autoimmune TCR bound to a myelin basic protein self-peptide (MBP_87–99_) and a multiple sclerosis-associated MHC class II molecule (HLA-DR2a), (**B**) close view of the docking site of (MBP_87–99_) peptide.

**Figure 4:**
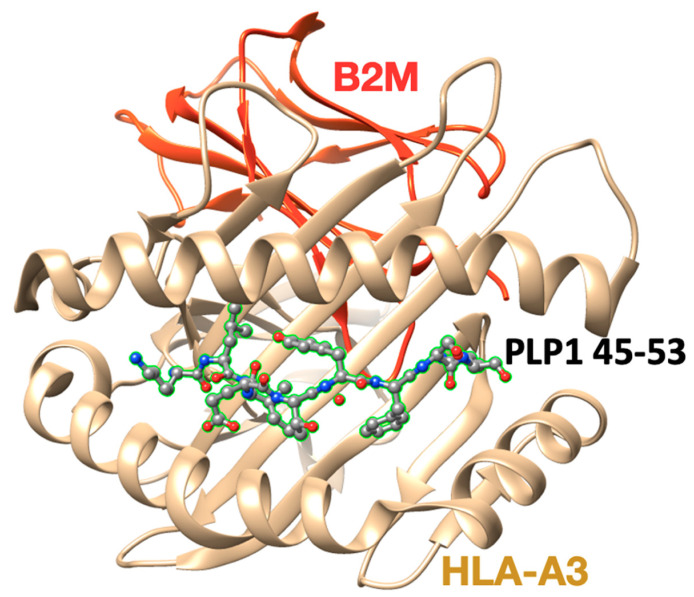
The X-ray crystal structure (pdb: 2XPG) of the human major histocompatibility (MHC) class I molecule HLA-A*0301 (HLA-A3) in complex with a PLP_45–53_ peptide.

Linear myelin epitopes MBP_83–99_, MBP_82–98_, MBP_85–99_, MBP_87–99_ (Figure 2 and Figure 3), MOG_35–55_, and PLP_139–151_ have been identified as agonist peptides inducing disease in humans and in animal models of MS. These peptides bind to MHC class II alleles, however, peptides binding to MHC class I have also been identified, primarily HLA-A*0301 (HLA-A3) in complex with a PLP_45–53_ peptide and the crystal structure known (Figure 4). Mutant analogs of linear agonist MHC class II peptides have been used to obtain information on the molecular basis of the disease. Data points out that mutant analogs of disease-associated epitopes can inhibit disease through two distinct mechanisms, one via the activation of antigen-specific regulatory T cells, or two, by activation and secretion of appropriate cytokines. The application of MBP_83–99_-based altered peptide ligands inhibits MBP-reactive T cell proliferation in vitro [30]; this is attributed to anti-inflammatory Th2 cytokine secretion by T cells, primarily IL-4 and IL-10. These obtained results point out that cytokine regulation is the major mechanism through which T-cell receptor (TCR)-specific CD4+ T cells regulate encephalitogenic and potentially other bystander Th1 cells. Thus, the modulation of cytokine secretion by auto-reactive T cells through peptide or non-peptide mimetics, even in longstanding autoimmune disease through cytokine therapy, might be beneficial therapeutically. This beneficial response is achieved by switching the function of myelin reactive T cells.

One of the first human clinical studies for patients with secondary progressive MS (MAESTRO-01) used the agonist MBP_82–98_ peptide (dirucotide). Intravenous injection of MBP_82–98_ peptide delayed disease progression. However, this effort was suspended at phase III stage since it lacked efficacy and did not meet primary endpoints [40,41].

Two mutant peptides namely [R^91^, A^96^]MBP_87–99_ and [A^91^,A^96^]MBP_87–99_ derived from the immunodominant agonist identified in MS (MBP_87–99_) were synthesized. The chosen mutations occurred at amino acids K^91^ and P^96^ as they are critical TCR contact sites. Immunization of mice with these altered peptide ligands (ALPs) emulsified in complete Freund’s adjuvant induced both IFNγ and interleukin-4 (IL-4) responses while the native MBP_87–99_ peptide induced only IFNγ responses. The linear MBP_72–85_ peptide (EKSERSEDENPV) is well known to induce experimental autoimmune encephalitis (EAE), and D^79^ mutation with A^79^ resulted in an analog that suppressed the induction of EAE [22]. In addition, the immunodominant peptide from PLP, HSLGKWLGHPDKF, is a naturally processed epitope. A double mutation of the agonist PLP_139–151_, in which both of the TCR binding sites are replaced with Leu or Arg ([L^144^, R^147^]PLP_139–151_), is able to antagonize PLP-specific T cell clones in vitro [42]. The mutated analog inhibited EAE and prevented clinical disease progression when administrated in the early stage of EAE induction [42]. Antibodies against the minor protein MOG have been noted in inflammation areas of MS. This proves that antibodies do play a role in MS and cooperate with antigen-presenting cells in myelin destruction. Blocking the effects of these MOG antibodies with secondary antibodies or non-peptide mimetics might be an important avenue for future therapy.

### 2.2. Cyclization of Selective Linear Epitopes and Their Derivatives

Linear peptide are known to be sensitive to proteolytic enzymes, which results in their degradation. Cyclization of linear peptides increases their stability in vitro and in vivo [43]. In an attempt to develop non-peptide mimetics, cyclic peptides are an important intermediate step towards this. As such, cyclic counterparts of linear peptides have been synthesized in an effort to improve their biological properties and structural stability [20,21,22,23,24,25,26]. Cyclic MBP_82–98_ exerts strong binding to the HLA-DR2 allele but has lower affinity binding to the HLA-DR4 allele. Cyclic analogues of dirucotide proved to be promising leads, and it is proposed that they be evaluated for their ability to alter T cell responses for therapeutic benefit against MS [40,41].

Spectroscopic data combined with Molecular Dynamics (MD) calculations showed that the linear MBP_72–85_ peptide adopts a pseudo-cyclic conformation. Based on this information, the cyclic analogue QKSQRSQDQNPV-NH_2_ was rationally designed. The cyclization of this molecule was achieved by connecting the side-chain amino and carboxyl groups of Lys and Glu at positions 2 and 9. This cyclic analogue exerted similar biological activity to the linear peptide; however, in EAE experiments, the cyclic analogue completely suppressed EAE by co-injection with the agonist peptide in Lewis rats. The similar potencies propose that cyclization does not substantially affect the conformational properties of its linear analogue and provides support to its proposed pseudo-cyclic conformation. In addition, this study proposes that a pseudo cyclic conformation for the MBP_72–85_ epitope allows D^81^ and K^78^ binding to the trimolecular complex MHC-peptide-TCR, and as a consequence, it inhibits EAE [23].

**Figure 5:**
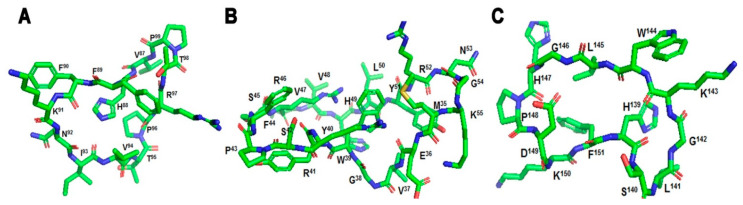
(**A**) cyclic-MBP_87–99_ (VHFFKNIVTPRTP), (**B**) cyclic-MOG_35–55_ (MEVGWYRSPFSRVVHLYRNGK), (**C**) cyclic-PLP_139–151_ (HSLGKWLGHPDKF).

A cyclic analogue, cyclo(87–99)MBP_87–99_ (Figure 5), of the human immunodominant MBP_87–99_ epitope, was synthesized based on the same rational as MBP_72–85_ epitope. This cyclic analogue in the same manner was shown to mimic the effects of the linear MBP_87–99_ epitope peptide, and thus to induce EAE, bind HLA-DR4, and increase CD4 T-cell line proliferation. The mutant cyclic peptides, cyclo(91–99)[A^96^]MBP_87–99_ and cyclo(87–99)[R^91^A^96^]MBP_87–99_, suppressed, to a varying degree, EAE, and possessed the following immunomodulatory properties: (i) they suppressed the proliferation of a CD4 T-cell line raised from a MS patient; (ii) they scored the best in vitro Th2/Th1 cytokine ratio in peripheral blood mononuclear cell (PBMC) cultures, inducing IL-10 selectively; (iii) they bound to HLA-DR4, first to be reported for cyclic MBP peptides; and (iv) they were found to be more stable to lysosomal enzymes and Cathepsin B, D, and H, compared to their linear counterparts. Such beneficial properties establish these synthetic peptides as putative immunotherapeutics for treating MS and potentially other Th1-mediated autoimmune diseases [23]. The mutations have been chosen as they identified the major TCR contact sites by X-ray crystallographic studies of human MHC and Molecular Dynamics (MD) studies using murine MHC.

The cyclic-MOG_35–55_ peptide, cyclized at the C- and N-terminal amino acids (cyclic-MOG_35–55_), altered the 3D conformation of the linear MOG_35__–__55_ peptide (Figure 5). Following the injection of cyclic-MOG_35__–__55_ during disease induction, EAE, demyelination, and chronic axonopathy in acute and chronic phases of disease were reduced. Molecular docking and spectroscopic data revealed milder interactions between the cycic-MOG_35–55_ and mouse or human MHC class II alleles (H2-IA^b^ and HLA-DR2) [44]. Likewise, synthesis of cyclic PLP_139–151_ peptide and injection in SJL/J mice showed that cylic-PLP_139–151_ analog is minimally encephalitogenic when administered to induce EAE (Figure 5). In particular, cyclic-PLP_139–151_ analog showed low disease burden and minimal inflammatory, demyelinating, and axonopathic pathology compared to its linear counterpart. Proliferation assays confirmed the low stimulatory potential of the cyclic-PLP_139–151_ compared to linear PLP_139–151_ as well as the induction of lower antibody responses. Comparative molecular modeling studies between the two molecules may explain the biological data, as it was shown that different amino acids are involved in the TCR recognition [42]. It is clear that cyclic modification of linear peptide counterparts may provide novel approaches for future, immunomodulative treatments against MS.

### 2.3. Conjugation of Selective Epitopes with Mannan 

Mannan, a poly-mannose isolated from the cell wall of yeasts, has been shown to exert immunomodulatory effects in the cancer settings in vitro [45,46,47,48,49,50], in vivo (inbred mice, transgenic mice, rats, rabbits, and chickens) [51,52,53,54,55,56,57,58,59,60,61,62], in rhesus macaques [63,64,65], and in human clinical trials [66,67,68,69,70,71,72,73,74,75]. Mannan targets antigens to the mannose receptor, antigens endocytosed for MHC class I or II presentation, and modulation of appropriate T cells [45,53,54,55,76,77,78,79,80,81]. In relation to autoimmune disorders, mannan conjugates (i) represent a new class of immunoregulators that directly and selectively target a population of immune cells that are implicated in the pathogenesis and progression of disease; (ii) provide first line treatment that selectively tolerates or inactivates disease-inducing cells in patients and also prevents progression of disease by stopping diversification of the autoimmune response to additional epitopes; (iii) allows easier formulation of newly discovered molecules within the mannan matrix platform; and (iv) can achieve block-buster status as a global vaccine drug for efficient treatment of MS [27,28,29,30].

Altered peptide ligands, where one to two amino acid mutations are made to those interacting with the TCR are able to alter an agonist peptide into an antagonist peptide by reduction of hydrogen bond interactions [82]. Cyclization of peptides allows for their stronger stability and protection against enzymatic and proteolytic degradation [43]. As such, a cyclic APL, cyclo(87–99)[A^91^,A^96^]MBP_87–99_] reduced Th1 responses, but when conjugated to reduced mannan, an additional significant reduction of Th1 responses and moderate Th2 responses was induced (Table 1) [27,30]. Likewise, APL of linear and cyclic MBP_83–99_ analogs, MBP_83–99_(A^91^,A^96^), conjugated to reduced mannan, resulted in diversion of Th1 response to Th2 [27]. The use of reduced mannan to further divert immune responses to Th2 when conjugated to MBP peptides constitutes a novel strategy for immunotherapy of the disease. The main advantages of mannan conjugates is their stability and non-toxicity. In addition, linear and cyclic peptide analogs based on MBP_83–99_ immunodominant epitope conjugated to reduced mannan via (KG)_5_ or keyhole limpet hemocyanin (KLH) linkers, were evaluated for their biological/immunological profiles in SJL/J mice. Of all the peptide analogs tested, linear MBP_83–99_(F^91^) and MBP_83–99_(Y^91^) conjugated to reduced mannan and cyclic MBP_83–99_(F^91^) conjugated to reduced mannan yielded the best immunological profile and constitute novel candidates for further immunotherapeutic studies against MS for translation into human clinical trials. Immune responses were diverted from Th1 to Th2 in SJL/J mice and generated antibodies that did not cross-react with native MBP protein. Molecular modeling was used to identify H-bonding and van der Waals interactions between peptides and MHC (I–A^s^) [21]. Furthermore, MBP_87–99_(R^91^, A^96^) conjugated to reduced mannan induced 70% less IFNγ compared with the native MBP_87–99_ peptide. However, MBP_87–99_(A^91^,A^96^) conjugated to reduced mannan did not induce IFNγ-secreting T cells, elicited very high levels of IL-4, and antibodies generated did not cross-react with the native MBP_87–99_ peptide (Table 1). It is clear that this double-mutant peptide analog conjugated to reduced mannan is able to divert immune responses from Th1 to Th2 and is a promising mutant peptide analogue for use in studies exploring potential treatments for MS [21].

In studies by Tseveleki et al. [28], MOG_35–55_ peptide conjugated to mannan in oxidized or reduced forms protected mice against EAE in prophylactic and therapeutic protocols, with oxidized-conjugated peptides giving the best results. Protection was peptide-specific and associated with reduced antigen-specific T cell proliferation. Mannan-MOG_35–55_ peptide pulsed bone marrow-derived dendritic cells (DC) showed up-regulated expression of co-stimulatory molecules and induced active T cell tolerance, suppressing ongoing EAE; whilst mannan-MOG_35–55_ peptide vaccinated mice did not reduce the proliferation of transferred MOG-specific T cells. MOG_35–55_-specific T cells cultured with mannan-MOG_35–55_-loaded DCs showed reduced proliferation and equal Th1 and Th17 cell differentiation as those with MOG_35–55_-loaded DC. Results show that mannan-conjugated myelin peptides protect mice against EAE through the expansion of antigen-specific Th1 and Th17 cells with impaired proliferation responses and DC-induced co-stimulatory signals that are required for licensing them to become fully pathogenic T cells [28]. In another study by Dagkonaki et al. [29], CNS autoantigens conjugated to oxidized mannan were shown to induce antigen-specific T cell tolerance and protection against EAE in mice (Table 1). The results showed that peptides conjugated with mannan induce peripheral type 2 myeloid cell responses and T cell anergy, and suggests that mannan-peptide conjugates may be useful for suppressing antigen-specific CD4+ T-helper cell responses in the context of human autoimmune CNS demyelination [29].

Further, the immunogenicity of linear (Cit^91^,A96,Cit^97^)MBP_87–99_ and its cyclic analog-cyclo(87–99)(Cit^91^,A96,Cit^97^)MBP_87–99_ when conjugated to mannan were studied in SJL/J mice. It was found that mannosylated cyclic citrullinated APL induced stronger T cell proliferative responses and IFNγ cytokine secretion in respect to its linear counterpart [25].

### 2.4. Citrullination of MBP Derived Peptides Role in Disease

Citrullination is a post-translational modification of peptidyl-arginine that plays a role in normal functioning of the immune system and also suspected to play a pathophysiological role in MS [83,84,85,86,87,88,89]. Citrullinated proteins of MBP are present in white matter lesions in the central nervous system in MS ([25,83,84,85,86,87,88,89]). Linear [Cit^91^,A^96^,Cit^97^]MBP_87–99_ that resulted from citrullination of 91,97 Arg residues in linear antagonist [R^91^,A^96^]MBP_87–99_ were synthesized and evaluated on PBMC from patients with MS and controls. Both peptides caused a Th1 polarization in all MS patient PBMC cultures. Culture with a non-citrullinated MBP peptide results in heterogeneous cytokine secretion that differs between individual patients. In addition, cyclo(87-99)[Cit^91^, A^96^, Cit^97^]MBP_87–99_ shows similar properties to the linear counterpart [25]. Thus, citrullination of self-antigens may potentially trigger disease in susceptible individuals. This result triggers future research to new substances that inhibit citrullination and arrest epitope spreading and worsening of MS [25]. 

### 2.5. Non-Peptide Mimetic Analogs

**Figure 6:**
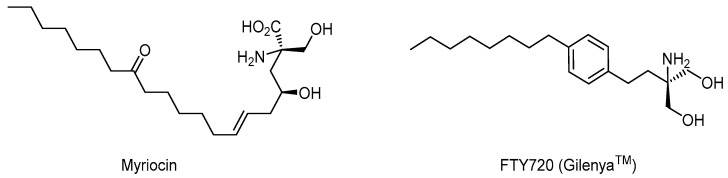
Structures of Myriocin (**left**) and FTY720 (**right**).

**Figure 7:**
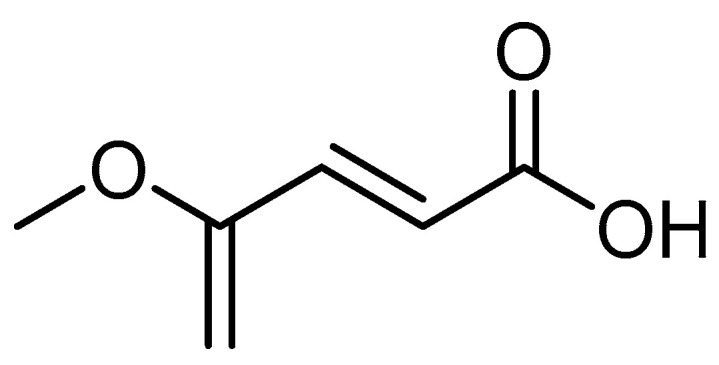
Structure of monomethyl fumarate.

Different molecules such as the fumaric acid and its esters are reported to be promising for MS treatment, exerting an antioxidative mechanism of action [31]. FTY720 (Gilenya^TM^) is derived from the natural product myriocin and constitutes the first oral treatment for MS [32] (Figure 6). Recently, monomethyl fumarate (MMF, Bafiertam) was reported to be well tolerated, safe, and effective [33] (Figure 7).

**Figure 8:**
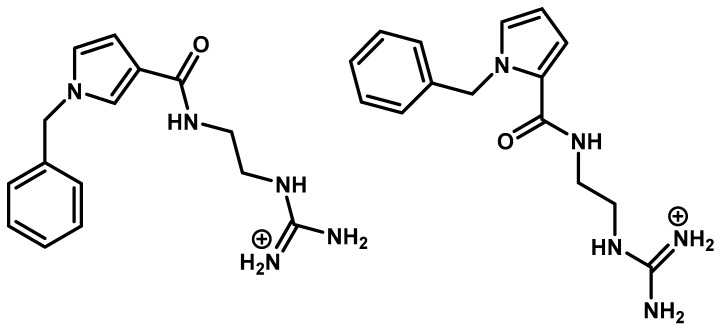
The most promising non-peptide inhibitors derived from in silico studies proved to have bioactivity against EAE.

Searching the literature, there is only one publication that claims the discovery of non-peptide mimetic molecules derived directly from peptides. The example is referred to the conformational analysis of MBP_83–96_ epitope. The authors searched for molecules that inhibit the trimolecular complex formation and consequently the proliferation of activated T-cells. They generated a structure-based pharmacophore and used ZINC as a chemical database to extract candidates. They used semi-empirical and density functional theory methods to predict the binding energy between the proposed non-peptide mimetics and the TCR. They decided, using this analysis, to synthesize six molecules. From these molecules, the following two were the most promising as they inhibited the stimulation of T-cells by the immunodominant MBP_83–99_ peptide from immunized mice (Figure 8) [34].

### 2.6. Nanotechnology Approaches

In the light of nanotechnology development, the approaches of discovering peptides linear or cyclic in structure or conjugated with oxidized or reduced mannans can be improved. Drug delivery vehicles can be developed that can selectively drive these simple or conjugated peptides to their site of action. Examples are mentioned in the literature for such application. A bifunctional peptide (small and soluble) and PLGA NPs (large and insoluble) resulted in relative greater EAE suppression [31,32,33,34,35,36,37,38,39].

In a recent review article, the recent advances in nanomedicines for MS therapy were outlined [35]. Nanomaterials have the potential to carry therapeutic agents through the blood brain barrier towards the lesion sites and can promote the remyelination process, achieving neuroprotection and neurodegeneration. In addition, they can act as effective ingredients for stem cell therapies. There is an intense effort to develop nanomedicines for clinical trials by reducing their toxicity and adverse effects [90].

In another recent article entitled “Nanocariers as potential drug delivery candidates for overcoming the blood-brain barrier: challenges and possibilities” [39] the different nanosystems and their potential as drug carriers are outlined. An optimistic view is presented for the future of nanocarriers in the field of treatment of CNS-related disorders, as their ability to pass the blood brain barrier to deliver therapeutic doses of the drug, is very promising [90].

## 3. Future Aspects

**Figure 9:**
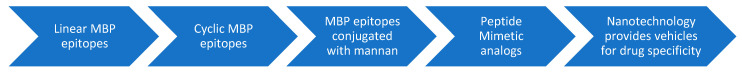
The road from linear epitope peptide to the therapeutic drug.

The development of an efficient treatment against MS has a long way to go. The existing therapies are not satisfactory. The multifactorial disease is difficult to be tackled. The different approaches mentioned herein will certainly aid in new and more efficient treatments. More efforts must be made for developing non-peptide analogs using rational design and the knowledge derived from the spectroscopic and computational chemistry studies on peptides. The cyclization of myelin epitope peptides and the conjugation of linear or cyclic peptides with mannan is a promising field that must be strengthened. Furthermore, nanotechnology has emerged to aid in this effort by providing vehicles that will lead potential drug leads specifically to the right target. The steps in the discovering of new leads to treat MS are illustrated below (Figure 9).

## 4. Conclusions

Linear MBP, PLP, and MOG epitopes were used as tools to study the molecular basis of MS. To increase their stability and enhance their pharmacological profile, linear peptides were cyclized. As such, cyclic-MOG and cyclic-PLP altered the conformation of the peptide, resulting in reduced EAE in animal models. In addition, one to two amino acid mutations were made to TCR contact residues (altered peptide ligands), which were shown to enhance Th2 anti-inflammatory responses over the predominant Th1 pro-inflammatory responses. However, this enhancement is not adequate, and as such, conjugation to mannan is required, which further shifts Th1 to Th2 responses. The future holds promise for novel immune modulators including cyclic peptides, altered peptide ligands, and the use of mannan to deliver the peptides to appropriate antigen-presenting cells.

## Figures and Tables

**Table 1:** Biological effects of myelin-derived peptides (linear, cyclic, mannan conjugated).

Peptide Analog [Reference]	Major Effects
MBP_83–99_ and PLP_139–151_ [10,11,12,13,14,15,16,17,18]	These agonist peptides are involved in the pathophysiology of MS and also induce EAE in animal models.
MBP_82__–__98_ [40,41]	Dirucotide in animal models inhibits disease and in early human clinical trials showed efficacy; however, the peptide did not meet primary endpoints in phase III-trials.
cyclic(87–99)[MBP_87__–__99_] [23]	Stimulates Th2 cytokines and inhibits EAE in mice.
MBP_87__–__99_(R^91^,A^96^), MBP_87__–__99_(A^91^,A^96^) [21]	Induces IL-4 and antagonizes IFNγ responses in mice.
MBP_72__–__85_ [22]	These agonist peptides induce EAE in mice and Th1 responses in humans.
MBP_72__–__85_(A^79^) [22]	Suppresses EAE in mice.
PLP_139__–__151_(L^144^, R^147^) [42]	Antagonizes PLP-specific T-clones in vitro.
cyclic-MBP_82__–__98_	Exerts strong binding to the HLA-DR2 and lowers binding to the HLA-DR4 allele in vitro
cyclic-MBP_87__–__99_(A^96^) or (R^91^A^96^) [21,30]	Suppresses proliferation of CD4+ T cells and exerts IL-10 selectivity in vitro. Binds to HLA-DR4 and is stable to lysosomal enzymes and cathepsins B, D, and H.
cyclic-MOG_35__–__55_ [44] cyclic-PLP_131__–__151_ [42] linear and cyclic-MBP_83__–__99_(A^91^,A^96^) [27] Mannan-linear and cyclic-MBP_83__–__99_(A^91^,A^96^) [27]	Reduces EAE, demyelination, and chronic axonopathy in acute and chronic phases of EAE in mice. Low disease burden in regards to EAE in mice with minimal inflammatory, demyelinating, and axonopathic pathology compared to its linear counterpart. Decreases IFNγ responses in mice. Diverts the “bad” IFNγ to “good” IL-4 cytokine in mice.
Mannan-MOG_35__–__55_ [28]	Protects mice against EAE in prophylactic and therapeutic protocols, with oxidized-conjugated peptides giving the best results.
Cyclo(87–99)MBP_87-99_(A^91^,A^96^) [27,30] Mannan-cyclo(87–99)MBP_87-99_(A^91^,A^96^) [27,30]	Decreases Th1 responses. Shifts Th1 responses to Th2 responses.
MBP_87__–__99_[Cit^91^,A^96^,Cit^97^] [25] cyclic-MBP_87__–__99_[Cit^91^,A^96^,Cit^97^] [25]	Induces T-cell proliferation and IFNγ secretion in mice. Activates T cells and increases IFNγ secretion in mice.

## Data Availability

The data presented in this study are available on request from the corresponding author.
